# Advances in the Synthesis of Crystalline Metallosilicate Zeolites via Interlayer Expansion

**DOI:** 10.3390/molecules26195916

**Published:** 2021-09-29

**Authors:** Chaoqun Bian, Yichang Yang, Xiaohui Luo, Wenxia Zhang, Jie Zhang, Longfeng Zhu, Jianping Qiu

**Affiliations:** 1 Pharmaceutical and Material Engineering School, Jinhua Polytechnic, Jinhua 321000, China; jhc0579@163.com (X.L.); 0420@163.com (W.Z.); 0579zjzj2@sina.com (J.Z.); 2 College of Biological, Chemical Sciences and Engineering, Jiaxing University, Jiaxing 314001, China; 2112001238@zjut.edu.cn; 3 College of Chemical Engineering, Zhejiang University of Technology, Hangzhou 310014, China; 4 Xingzhi College, Zhejiang Normal University, Jinhua 321004, China; jipiqi@126.com

**Keywords:** layered silicates, interlayer expansion, zeolites, synthesis, metal heteroatoms insertion

## Abstract

Given the numerous industrial applications of zeolites as adsorbents, catalysts, and ion-exchangers, the development of new zeolite structures is highly desired to expand their practical applications. Currently, a general route to develop new zeolite structures is to use interlayer expansion agents to connect layered silicates. In this review, we briefly summarize the novel zeolite structures constructed from the lamellar precursor zeolites MWW, RUB-36, PREFER, Nu-6(1), COK-5, and PLS-1 via interlayer expansion. The contents of the summary contain detailed experiments, physicochemical characterizations, possible expansion mechanisms, and catalytic properties. In addition, the insertion of metal heteroatoms (such as Ti, Fe, Sn) into the layered zeolite precursor through interlayer expansion, which could be helpful to modify the catalytic function, is discussed.

## 1. Introduction

In the past decades, a variety of novel zeolite structures have been synthesized, thus employing the practical application widely in industrial production as adsorbents, catalysts, and ion-exchangers, which has produced great economic benefits [1,2,3,4]. The synthesis of zeolite structures with new topologies is of constant interest from the viewpoint of both fundamental research and industrial application s [5,6,7,8]. In most cases, three-dimensional (3D) zeolites with 4-connected framework form directly from inorganic precursors assembled in the presence of organic or inorganic structure-directing agents (SDAs) [9,10,11]. In addition, two-dimensional (2D) layered zeolites with new topological structures, which consist of lamellar sheets because the bridging units between the layers are not connected with four T-atoms but only two, operated by calcination [12], swelling [13,14], delamination [15,16], and interlayer expansion [17,18,19,20], are considered as promising catalysts for many catalytic reactions. Table 1 lists published zeolite synthesis via topotactic condensation of layered silicates. The close names of zeolites related to nearly identical materials. These new topological structures not only maintain the basic structural unit of the zeolites but also offer larger pore size and higher specific surface area, making them more suitable for the reaction of molecules of different sizes, thereby broadening the range of available zeolites [21,22].

Interlayer-expansion zeolites (IEZs) usually use silylating agents or metal salt to connect layer silicates to novel 3D frameworks [35,36,37,38], which has led to several new microporous zeolite frameworks, such as ferrierite (FER) from PREFER, the MWW-type framework (MWW) from MCM-22 [30,38], COE-3 (CDO) from RUB-36 [39], and COE-5 (MFS) from COK-5 [40]. As the number of microporous zeolite frameworks increases, IEZs are gradually forming an important family of microporous materials [41,42,43,44,45,46]. The silylating agents generally include a single silicon dichlorodimethylsilane (DCDMS), dihydroxydimethylsilane (DHDMS), and diethoxydimethylsilane (DEDMS), and two silicon macromolecular silane reagents 1,2-dichlorotetramethyldisilane (ClMe_2_Si-SiMe_2_Cl) and 1,3-Dichlorotetramethyldisiloxane (ClMe_2_Si-O-SiMe_2_Cl). The silane agents provide a Si source for the interlayer space with the replacement of the organic SDA. Notably, the insertion of Si atoms increases the pore size of the original materials, which in turn increases the scope of applications of these materials [44,46,47,48]. For example, the well-known interlayer expanded MWW-type aluminosilicate has been found to play an important role in the selective production of bulk petrochemicals [31,32,42,48].

In addition, the insertion of metal heteroatoms (such as Ti, Fe, Sn) has also been reported [47,48,49,50,51], which could be beneficial for modifying the catalytic function. One solution is to add heteroatoms during the synthesis of the precursor. For example, the microporous titanosilicate Ti-COE-4 zeolite is synthesized by interlayer expansion of Ti-RUB-36 with DHDMS [50]. Another novel synthesis route involves using metal salts is to connect layered silicates instead of normal silylating agents. A typical example of this route is Fe-IEZ-RUB-36, which is synthesized by interlayer expansion of RUB-36 with FeCl_3_ [51].

For readers not familiar with layered zeolites, only one typical sample in a series of similar layer materials is discussed in this review (RUB-36 ≈ PLS-4 ≈ UZM-13 and RUB-18 ≈ ITQ-1 ≈ MCM-22 ≈ EMM-10). Herein, we briefly summarize the novel zeolite structures constructed from the lamellar precursor zeolites MWW, RUB-36, PREFER, Nu-6(1), COK-5, and PLS-1 via interlayer expansion (Table 2). The summary includes a discussion of experiments, physicochemical characterizations, possible expansion mechanisms, and catalytic properties. To be consistent with the literature and simplify the discussion, the IEZs are named as IEZ-ABC, M-IEZ-ABC, IEZ-Me-ABC, and M-IEZ-Me-ABC, where “ABC” is the code of the original zeolite structure, M is the metal introduced into the zeolite by interlayer expansion, and Me is the metal from the original layered zeolite. The layered precursor zeolites and IEZs are listed in Table 2, along with the insertion agents. Furthermore, the ADOR (assembly-disassembly-organisation-reassembly) method is referenced as a special type of interlayer expansion.

## 2. Experiment

### 2.1. Synthesis of Layered Precursor Zeolites

The zeolite precursor RUB-36 was synthesized from an initial gel with a composition of 1.0 SiO_2_:0.5 diethyl dimethyl ammonium hydroxide (DMDEAOH):10 H_2_O in an autoclave for 10–14 days at 140 °C [52]; The Me-RUB-36 was synthesized from a starting gel with 1.0 SiO_2_:0.005–0.006 MeO_2_:0.5 DMDEAOH: 10–13 H_2_O for 10 days at 140 °C [50,53]; For the synthesis of the COK-5 zeolite, it is necessary to use N,N,N,N′,N′,N′-hexamethyl pentane diammonium (Et_6_-diquat-5) as an organic SDA. Then, COK-5 is hydrothermally synthesized from a starting aluminosilicate gel with a composition of 1.0 SiO_2_:0.015 Al_2_O_3_:0.097 Na_2_O:0.105 SDA:41 H_2_O at 160 °C for 132 h [58,59]; The Me-MWW precursor (where Me = Ti, Al, Ga, Fe, etc.) was hydrothermally synthesized from the gel with a composition of 1.0 SiO_2_:0.039 MeO_2_:1.4 SDA:19 H_2_O or SiO_2_:(0–0.039) TiO_2_:0.67 B_2_O_3_:1.4 SDA: 19 H_2_O at 140 °C for 7–10 days under a rotation condition (100 rpm), where the organic SDA is hexamethyleneimine [60,61]; The FER lamellar precursor, so-called PREFER, was hydrothermally synthesized by using 4-amino-2,2,6,6-tetramethylpiperidine as an organic SDA. And the gel with the composition of 1.0 SiO_2_:1.0 SDA:1.5 NH_4_F:1.0 HF:15 H_2_O is crystallized in an autoclave at 170 °C for seven days [29]; PLS-1 samples are converted from the mixture with a composition of 1.0 SiO_2_:0.0989 TMAOH:0.268 KOH:3.4 1,4-dioxane:14.5 H_2_O crystalized at 150 °C for 10 days [47].

After crystallization, the solid product was filtered, washed, and dried at room temperature. The above as-synthesized precursors are calcined in air at 550 °C for 10 h to remove occluded organic SDA and thus form the corresponding zeolites with 3D structure.

### 2.2. Synthesis of IEZ-ABC Zeolites

The zeolite precursors are alkoxy silylated with interlayer-expansion agents in acidic medium. Typically, 1 g RUB-36, 0.13 g DCDMS, and 100 mL 0.1 M HCl are mixed and then transferred to an autoclave at 180 °C for 24 h. The white powder obtained is named as IEZ-RUB-36. The samples obtained after calcination are named as IEZ-CDO (the calcined RUB-36 samples are CDO topology). Similarly, IEZ-Me-CDO samples are obtained by using the same procedure as IEZ-CDO but with IEZ-Me-CDO samples.

Metal cations could be introduced at the linker sites by applying the similar synthesis procedure in the following. Typically, M-IEZ-CDO (M = Sn, Fe, Zn, etc.) is synthesized directly from RUB-36 in acidic conditions in the presence of the corresponding metal salt instead of silane reagents. For example, to get Fe-IEZ-CDO, 0.3 g of FeCl_3_·6H_2_O, 0.7 g of RUB-36, and 12.5 g of 0.3 M HCl, they undergo reaction in an autoclave for 24 h at 180 °C. M-IEZ-Me-CDO samples are obtained by the same procedure as used for M-IEZ-CDO.

## 3. Physicochemical Characterizations

### 3.1. Investigation of Interlayer Expansion with XRD and N_2_ Adsorption

Interlayer-expansion agents with two methoxy groups are chosen to connect two adjacent layers, possibly by reacting with surface silanol groups. Various zeolitic lamellar precursors are interlayer-expanded with organic silanes in acidic media into highly ordered 3D crystalline zeolite materials. 2D lamellar precursors have special layered silicates considered as high silica hydrous layered silicates. The general structure of the layers consists of terminal Si-OH or Si-O groups sticking out into the inter-layer space. Organic SDA between the layers separates the silica layers. The calcination of lamellar precursors removes the organic SDA and thus condenses the high silica hydrous layered silicates [62]. The interlayer-expansion agents insert extra atoms between the neighboring layers, which increases the layer distance [63,64]. Therefore, the IEZs do not condense after calcination.

**Figure 1:**
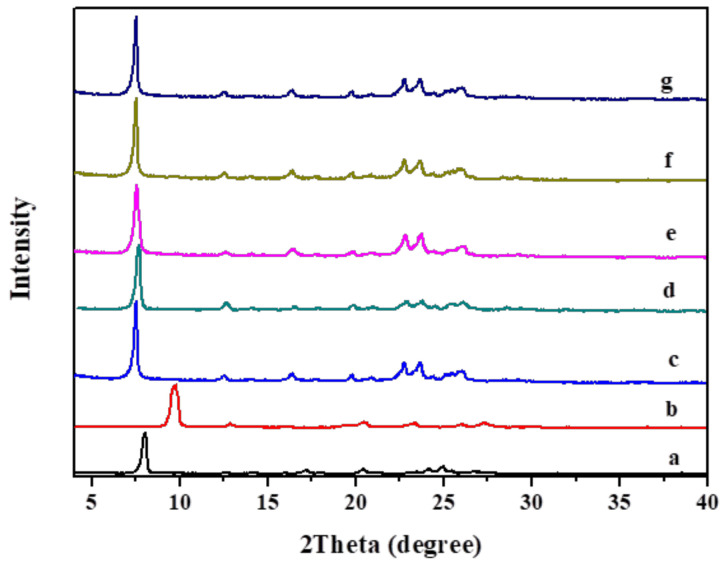
XRD patterns of (**a**) RUB-36, (**b**) CDO zeolite, (**c**) IEZ-RUB-36, (**d**) IEZ-CDO, (**e**) IEZ-Al- RUB-36, (**f**) Fe-IEZ-RUB-36, and (**g**) Fe-IEZ-Al-RUB-36. Reprinted with permission from ref. [49]. Copyright 2020, MDPI.

X-ray diffraction (XRD) reveals the layered crystal structures. Figure 1a–d shows XRD patterns of RUB-36 and IEZ-RUB-36 before and after calcination [49,52]. The layer-related diffraction of CDO (Figure 1b) shifts to higher 2*θ* compared with RUB-36 (Figure 1a), because RUB-36 is converted to the corresponding 3D crystalline structures after calcination. After treatment with DCDMS in acidic conditions, the IEZ-RUB-36 samples obtained showed a lower 2*θ* degree (from 8.14° to 7.53°, see Figure 1c). The calcined sample IEZ-CDO remains at a lower angle compared with CDO, which means an increase in layer spacing. This phenomenon is due to the insertion between the layers of the silicon species from DCDMS. This species might react with the high silica hydrous layered silicates on the silicate layers and thereby connect neighboring layers, leading to turn the 8-MR of CDO into the 10-MR of IEZ-CDO. The Si-O-Si groups do not condense during calcination. Finally, the *d* spacing increases from 9.2 to 11.7 Å, as shown in the Table 3.

Very interestingly, the incorporation of Al or Ti atoms into the CDO framework occurs in the original lamellar precursor synthesis. Al-RUB-36 or Ti-RUB-36 is incorporated into the RUB-36 framework via direct synthesis with addition of an Al or Ti source. And the interlayer expansion with DCDMS or DEDMS leads to the formation of IEZ-Al-RUB-36 [53] (Figure 1e) or IEZ-Ti-RUB-36 [50]. Afterward, IEZ-Al-CDO or IEZ-Ti-CDO would be obtained after calcination. In addition, replacing silylated agents by FeCl_3_, TiCl_3_ or metal-acetylacetone during interlayer expansion of precursors results in M-IEZ-Me-ABC. For example, Fe-IEZ-RUB-36 and Fe-IEZ-Al-RUB-36 [51,65] (Figure 1f,g) are obtained by interlayer expansion of RUB-36 or Al-RUB-36 with FeCl_3_, respectively. The Fe-IEZ-CDO and Fe-IEZ-Al-CDO samples obtained after calcination show a similar shift with a *d* spacing of 11.7 Å (Table 2), indicating the similarity of the crystal framework obtained.

N_2_ adsorption is a powerful tool to characterize the surface area and microporous volume of the samples. Table 3 lists the parameters of CDO and related samples [49,50,51,52,53,65]. The original layer samples, RUB-36, have a small microporous volume. The microporous volume increases from 0.12 m^3^/g for CDO to 0.131 m^3^/g for IEZ-CDO after interlayer expansion (Table 2), which has also been confirmed by the result of XRD patterns. In addition, the surface area also increases due to the increased pore size. Of course, M-IEZ-CDO and IEZ-Me-CDO have similar change trends [51,65].

**Figure 2:**
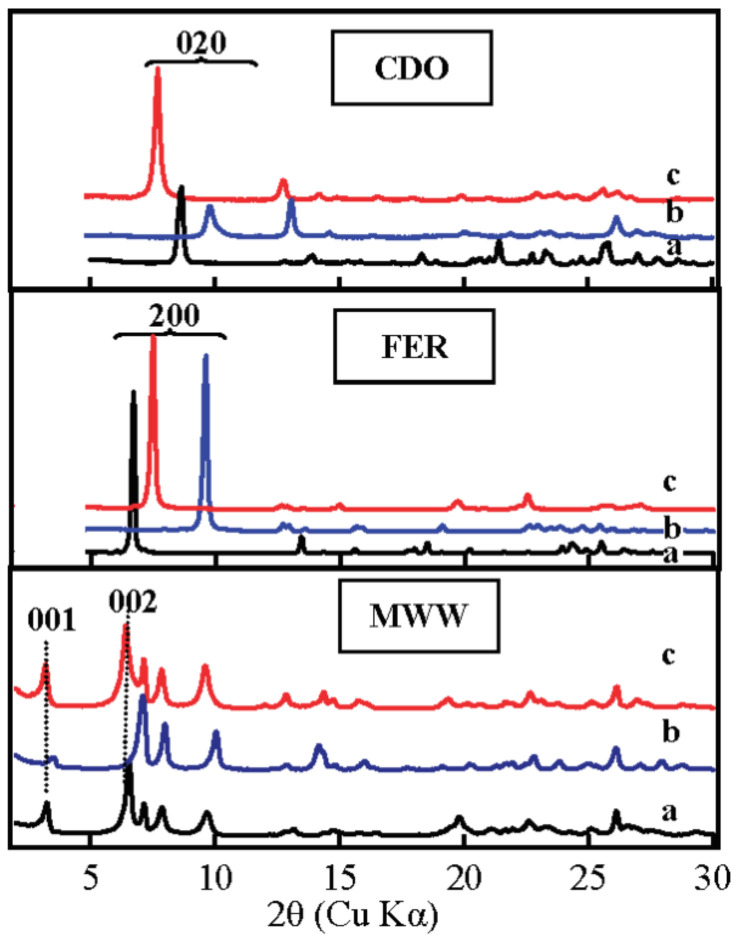
XRD patterns of (**a**) lamellar precursors, (**b**) 3D zeolites formed by the direct calcination of precursors, and (**c**) IEZs samples after further calcination. Reprinted with permission from ref. [36]. Copyright 2008, American Chemical Society.

The same scenario occurs in the synthesis of IEZ-Me-MWW (Figure 2). The zeolite IEZ-Ti-MWW is obtained by interlayer expansion of the precursor Ti-MWW [36,48,66]. Upon treatment in an acidic solution, the soluble silicon species from the zeolite crystals probably connect the Si-OH between adjacent layers. The 10-MR in the Ti-MWW precursor converts into a 12-MR ring in IEZ-Ti-MWW, which increases transport rates and the potential to filter a larger product. PREFER with 8-MR turns into IEZ-FER with 10-MR upon silylation with DEDMS (Figure 2). The IEZs synthesis strategy also applies to a variety of lamellar precursors [67,68], such as IEZ-NSI from Nu-6(1) [54], IEZ-MFS from COK-5 [40], IEZ-FER from PREFER [56], and IEZ-CDS-1 from PLS-1 [47] (Table 2).

**Figure 3:**
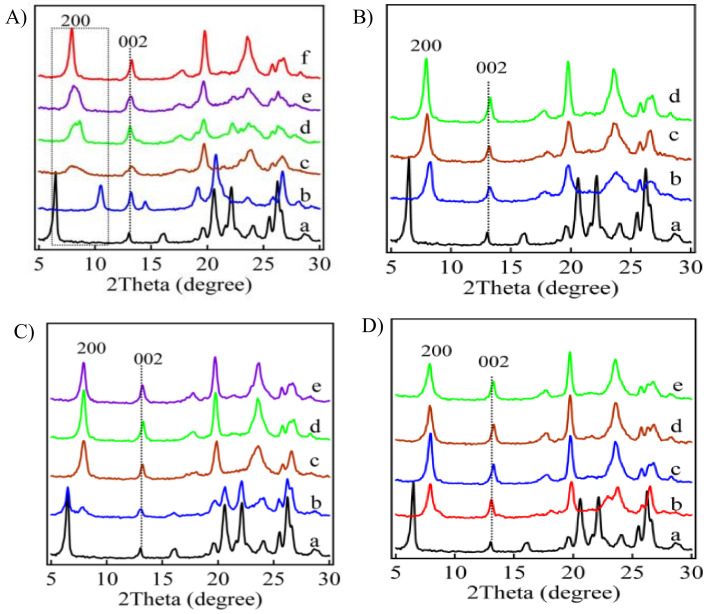
XRD patterns of (**a**) Nu-6(1) precursor (all silica), (**A**–**b**) NSI sample, and (from **A**–**c** to **D**–**e**) IEZ-Nu-6(1) under different conditions. The main conditions consist of 0.5 g Nu-6(1), 0.08 g DEDMS, 473 K, 2 M HCl solution, for a duration of 24 h. (**A**) XRD patterns for various solution compositions: (**A**–**c**) water, (**A**–**d**) ethanol/H_2_O (1:1), (**A**–**e**) ethanol/H_2_O (2:1), (**A**–**f**) ethanol. (**B**) XRD patterns for various temperatures: (**B**–**a**) room temperature, (**B**–**b**) 403 K, (**B**–**c**) 443 K, (**B**–**d**) 473 K. (**C**) XRD patterns for various acid concentrations: (**C**–**b**) 0.5 M HCl, (**C**–**c**) 1 M HCl, (**C**–**d**) 2 M HCl, (**C**–**e**) 3 M HCl. (**D**) XRD patterns for various amounts of DEDMS: (**D**–**b**) 0 g, (**D**–**c**) 0.08 g, (**D**–**d**) 0.13 g, and (**D**–**e**) 0.18 g. Reprinted with permission from ref. [54]. Copyright 2013, American Chemical Society.

To obtain optimal conditions, Wu et al. studied the factors that affect interlayer expansion by silylation. Silylating the Nu-6(1) precursor with DEDMS produces IEZ-Nu-6(1) [54]. Compared with the 3D NSI zeolite (curve b of Figure 3a), the calcined samples IEZ-NSI have a lower 2*θ*, as seen in Figure 3. Although the (200) diffraction undergoes the same shift under different conditions, the strength of the peak indicates a different level of response. As seen in Figure 3a, the (200) diffraction is relatively broad and low in intensity when silylation is done in an aqueous solution. Interestingly, this condition is always acceptable for other precursors such as RUB-39 or RUB-36. As opposed to other precursors [69,70], increasing the volume of ethanol improves the order of IEZ-Nu-6(1). In addition, Figure 3b shows that the higher temperatures are favorable for constructing well-ordered structures with interlayer-expanded pores. Furthermore, Figure 3c shows that the suitable acid concentration also leads to well-ordered structures. When HCl concentration is low (0.5 M), it produces a mixture of Nu-6(1) precursor and IEZ-Nu-6(1); however, increasing the acid concentration above 2 M HCl partly dissolves the samples in the solution.

Figure 3D shows how the amount of silane affects the structural order of IEZ-Nu-6(1). All of the (200) peaks undergo the same shift as a function of the amount of DEDMS (Figure 3D, curves b–e), which means that the similar interlayer expanded structure is obtained independent of the amount of silylated agent. The interlayer expanded structure is constructed even in the absence of DEDMS silane (Figure 3D, curve b) but it is of ow crystallinity. Similar phenomena have been reported for the direct acid treatment of post-synthesized Ti-MWW precursor [38,48]. Without interlayer expansion agents, soluble silicon species from the zeolite crystals probably serve as pillars to cross-link the layers.

**Figure 4:**
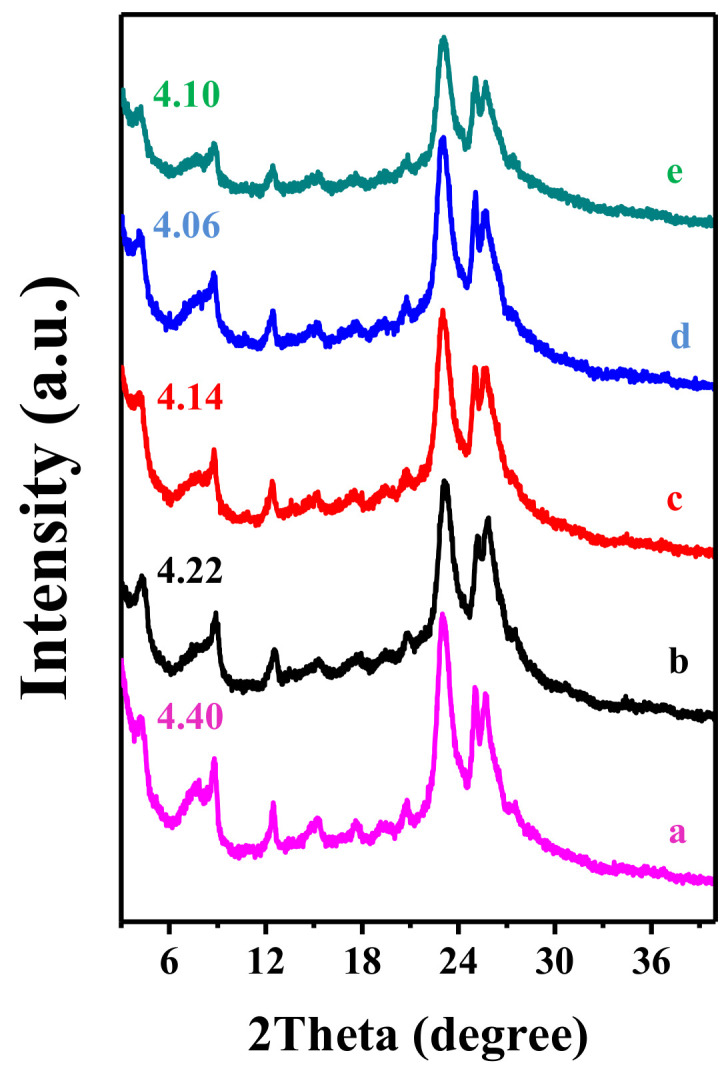
XRD patterns of (**a**) as-synthesized COK-5, (**b**) IEZ-COK-5-0, (**c**) IEZ-COK-0.185, (**d**) IEZ-COK-0.375, and (**e**) IEZ-COK-0.830. Reprinted with permission from ref. [40]. Copyright 2015, Elsevier.

In addition, it is worthy of note that, upon silylating the precursor COK-5 with DCDMS, the layer-related diffraction pattern shifts by a different degree (Figure 4) [40]. Samples with interlayers expanded with *x* g DCDMS per g COK-5 are denoted as IEZ-COK-5-*x* (*x* = 0, 0.185, 0.375, 0.830). When *x* is less than 0.375, the (001) peak shifts slightly to a lower angle. When *x* is more than 0.375, the peak remains at the same angle. This phenomenon might be caused by the layered structure with stacking disorder [59].

### 3.2. Investigation of Interlayer Expansion with Electron Microscopy

**Figure 5:**
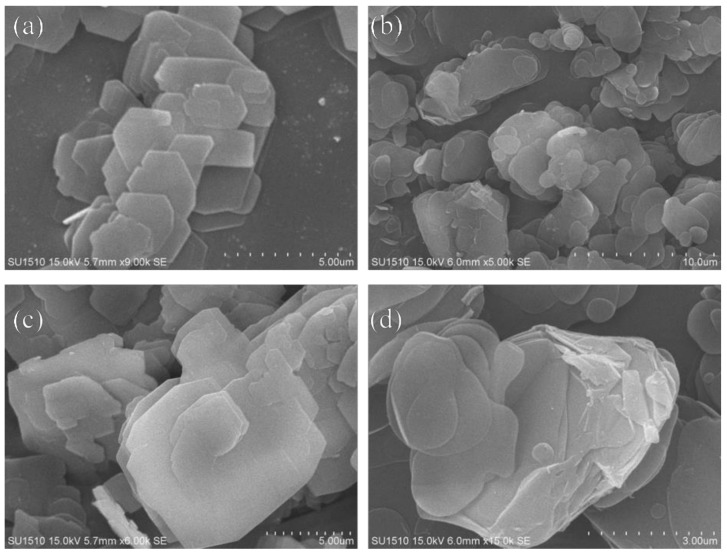
SEM images of (**a**) RUB-36, (**b**) IEZ-CDO, (**c**) Fe-IEZ-CDO and (**d**) Fe-IEZ-Al-CDO. Reprinted with permission from ref. [49]. Copyright 2020, MDPI.

The scanning electron microscopy (SEM) technique is usually used for revealing the crystal morphology. However, despite the IEZ reactions varying the separation between adjacent layers, the layer precursors and related IEZ materials retain a very similar morphology. Figure 5a–d show the SEM images of (a) RUB-36, (b) IEZ-CDO, (c) Fe-IEZ-CDO, and (d) Fe-IEZ-Al-CDO. All images exhibit a similar morphology with nanosheets. These results indicate that the interlayer expansion does not influence the sample morphology, which are similar with other references reported [36].

**Figure 6:**
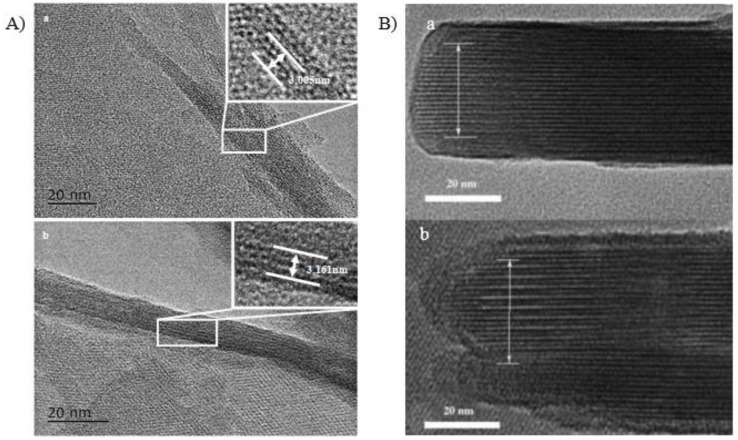
HRTEM images of (**A**)/(**a**) COK-5, (**A**)/(**b**) IEZ-MFS, (**B**)/(**a**) Ti-MWW, and (**B**)/(**b**) IEZ-Ti-MWW. Reprinted with permission from ref. [38,40]. Copyright 2004, Wiley and Copyright 2015, Elsevier.

High-resolution transmission electron microscopy (HR-TEM) could reveal the spacing between neighboring layers, which can provide direct evidence of the interlayer expansion. Figure 6A shows HRTEM images taken of the edge of the COK-5 and IEZ-COK-5 crystallites [40]. Three layers in IEZ-COK-5 spans are 3.161 nm (see inset in Figure 6A/b), which slightly exceeds the 3.005 nm spanned by three layers in COK-5 (see inset in Figure 6A/a), confirming that the interlayer expansion occurs along the *c* axis upon treating COK-5 with DCDMS. The same phenomenon could be observed in the interlayer expansion of Ti-MWW [38] (Figure 6B). The distance spanned by 10 layers in IEZ-Ti-MWW (Figure 6B/b) is comparable to that spanned by 11 layers in 3D Ti-MWW (Figure 6B/a), which reveals the expansion between neighboring layers in the former.

**Figure 7:**
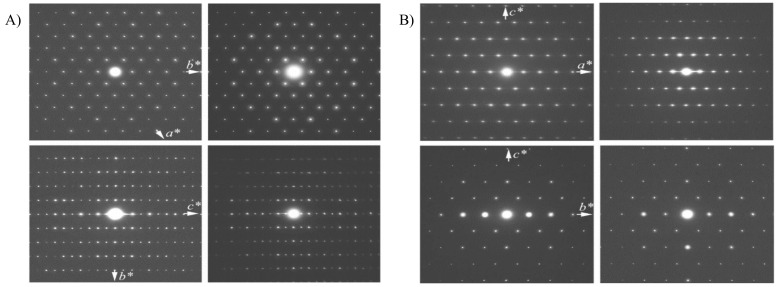
Selected-area electron diffraction patterns of (**A**) 3D MWW (left), IEZ-Ti-MWW (right) and (**B**) 3D-FER (left), IEZ-FER (right). Reprinted with permission from ref. [36]. Copyright 2008, American Chemical Society.

Figure 7A compares selected-area electron diffraction of 3D MWW and IEZ-Ti-MWW along two directions (100) and (001), and Table 4 lists the *d* spacings given by HRTEM between 3D MWW and IEZ-Ti-MWW [36]. The results show that IEZ-Ti-MWW has a pore array and pore size, which are remarkably similar with that of the 3D structure obtained by direct calcination in the layer. However, the well-ordered pores expand along the (001) direction from 25.3 to 28.2 Å. And this pore expansion is consistent with the XRD evidence that the pores enlarge from 10 to 12-MR. As shown in Figure 7B, the *d* spacing along the (200) direction also increases from 9.8 Å for 3D-FER to 12.1 Å for IEZ-FER. 

### 3.3. Investigation of Interlayer Expansion by Infrared Spectroscopy and Contact Angle

**Figure 8:**
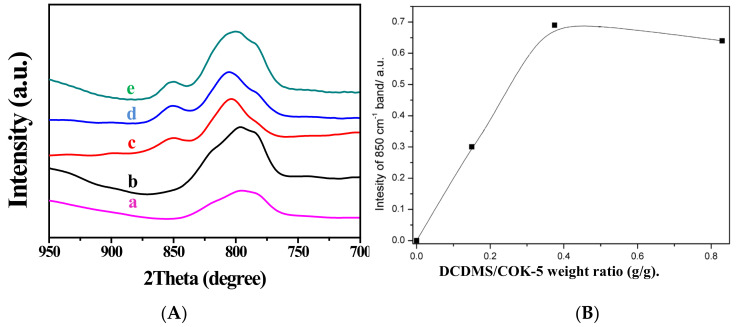
(**A**) IR spectra of (**a**) COK-5, (**b**) IEZ-COK-5-0, (**c**) IEZ-COK-5-0.185, (**d**) IEZ-COK-5-0.375, (**e**) IEZ-COK-5-0.830. (**B**) Intensity of 850 cm^−1^ band as a function of the amount of DCDMS used in the silylation of COK-5. Reprinted with permission from ref. [40]. Copyright 2015, Elsevier.

Infrared (IR) spectroscopy is always used to monitor the silylation of the lamellar precursors. With the as-synthesized lamellar precursors, an IR absorption peak appears in IEZ samples at 850 cm^−1^, which is assigned to asymmetric stretching of -CH_3_ groups attached to Si species [71,72,73], confirming the incorporation of the (CH_3_)_2_Si moiety into the zeolite. To investigate how the amount of silane affects the structural order, we study in detail the interlayer expansion of COK-5 with different amounts of silane as interlayer expansion agents. The intensity of the 850 cm^−1^ band increases with the increasing DCDMS amount, and then levels off at a silane-to-precursor weight ratio of 0.375 g/g (Figure 8), indicating that no further silane groups can be incorporated. The intensity of the 850 cm^−1^ band even decreases when the silane-to-precursor weight ratio exceeds 0.375 g/g, which is attributed to excessive silylation, reducing the pH of the solution and thereby hindering the further incorporation of Si(CH_3_)_2_ moiety. The above results are consistent with the XRD results.

**Figure 9:**
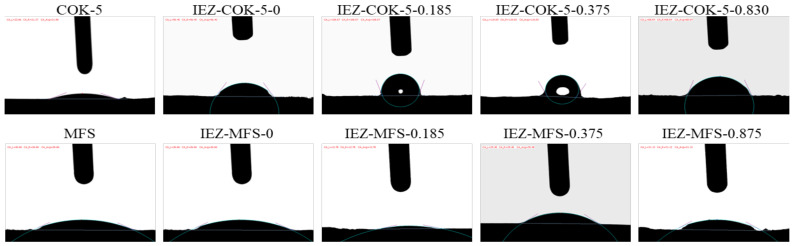
Contact angles of water on surface of COK-5, MFS, IEZ-COK-5, and IEZ-MFS produced using different amounts of DCDMS. Reprinted with permission from ref. [40]. Copyright 2015, Elsevier.

As known, most lamellar precursors currently studied are silicate crystals, and interesting phenomena occur in the aluminosilicate zeolite COK-5. When it is treated in an acidic solution, Al atoms from the aluminosilicate zeolite dissolve in the solution, and the increased Si/Al ratio decreases the wettability. Meanwhile, the interlayer expansion caused by the silylated agent introduces Si-(CH_3_)_2_ groups into the lamellar precursor, increasing the hydrophobicity. Table 5 and Figure 9 show the contact angles of water on the surface of COK-5, MFS, IEZ-COK-5, and IEZ-MFS produced with varying amounts of DCDMS [40]. The contact angle of COK-5 is about 22°, whereas calcined COK-5 (called MFS) produces a contact angle of 27°. This phenomenon might be related to the fact that calcination causes hydrophilic hydroxyl groups to condensate between layer and layer. When COK-5 zeolites are immersed in HCl solution without any DCDMS, the IEZ-COK-5-0 obtained produces a greater contact angle of 56°, which is attributed to the loss of Al atoms (Si/Al = 74), as confirmed by the Si/Al ratio. Moreover, calcined IEZ-COK-5-0 (called IEZ-MFS-0) produces a small contact angle of 27°, which is the same as the contact angle of MFS. This phenomenon is tentatively attributed to the loss of Al atoms, which has the same effect as the condensation of hydrophilic hydroxyl groups. Besides, the high Al content (the Si/Al ratio is as low as 11–12) can strongly impact the silylation for the leaching of framework Al species during interlayer expansion in a strongly acidic condition. The IEZ-Al-MWW could be obtained when the Si/Al ratio of Al-MWW is higher than 30 [36,74,75].

Upon treating COK-5 with DCDMS, the IEZ-COK-5 produced has an increased contact angle, which is attributed to the increased concentration of methyl groups in the zeolite framework [76,77,78]. The contact angle decreased when more than 0.375 g of DCDMS was added per gram of COK-5 because the extra DCDMS turns into HCl, which destroys aluminosilicate crystals. After calcination at 550 °C for 4 h, the contact angle of IEZ-MFS decreases, which is interpreted as a change in wettability due to methyl groups generated by calcination of hydrophilic silanols [77]. The contact angle of IEZ-MFS-0.185 goes to minimal value because the Si/Al ratio remains at 40, and the interlayer expansion incorporates the Si-OH groups into the zeolite framework. This phenomenon demonstrates the hydrophobic nature of the as-made IEZ materials. Clearly, the sample wettability may be tuned by inserting methyl groups and transforming methyl to silanol. Therefore, the contact angle of water can be tuned by adjusting the dosage of DCMDS.

### 3.4. Investigation of Interlayer Expansion by ^13^C and ^29^Si NMR Spectroscopy

**Figure 10:**
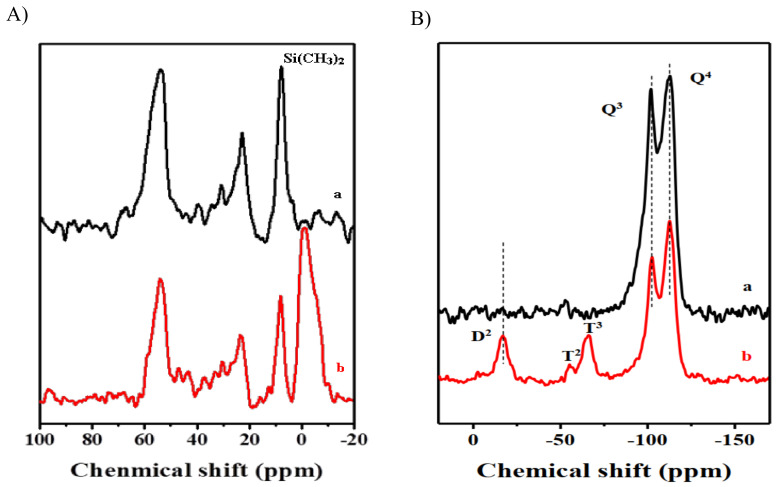
(**A**) ^13^C MAS NMR spectra and (**B**) ^29^Si MAS NMR spectra of (**a**) COK-5 and (**b**) IEZ-COK-5. Reprinted with permission from ref. [40]. Copyright 2015, Elsevier.

The incorporation of (CH_3_)_2_Si groups by the silylation of lamellar precursors is further investigated by ^13^C magic-angle spinning (MAS) NMR and ^29^Si MAS NMR techniques. Most silylation agents include Si(CH_3_)_2_ groups, which connect to Si-OH between adjacent layers. In contrast with the original lamellar precursor, the IEZ samples without further calcination retain the Si(CH_3_)_2_ groups. Using COK-5 and IEZ-COK-5 as an example, silylation produces a new resonance with the chemical shift at −2.0 ppm in the ^13^C MAS NMR spectrum (Figure 10A–b), which is due to the configuration of the Si(CH_3_)_2_ groups [73,79]. This result also confirmed by the IR spectra result. In addition, COK-5 produces two strong peaks with the chemical shift at −113 and −101 ppm in the spectra of ^29^Si MAS NMR, which are associated with the Si(SiO)_4_ (Q^4^) and (OH)Si(SiO)_3_ (Q^3^) species, respectively. Furthermore, IEZ-COK-5 produces an additional peak with the chemical shift at −17 ppm in the spectra of ^29^Si MAS NMR, which is attributed to the Si(CH_3_)_2_(SiO)_2_ (D^2^) species. The peaks with the chemical shift at −53 and −67 ppm are assigned to the Si(CH_3_)(OH)(SiO)_2_ (T^2^) and Si(CH_3_)(SiO)_3_ (T^3^) species. These results show that the Si(CH_3_)_2_ species is indeed inserted into the zeolite framework [80,81,82]. Notice in particular that, compared with COK-5, IEZ-COK-5 has a low concentration of the Q^3^ species, indicating that silylation occurs through the reaction of silane groups with the silanols on the layer surface to link neighboring layers to a microporous framework silicate with functionalized bridging linker groups [-O-Si(CH_3_)_2_-O-] [82].

**Figure 11:**
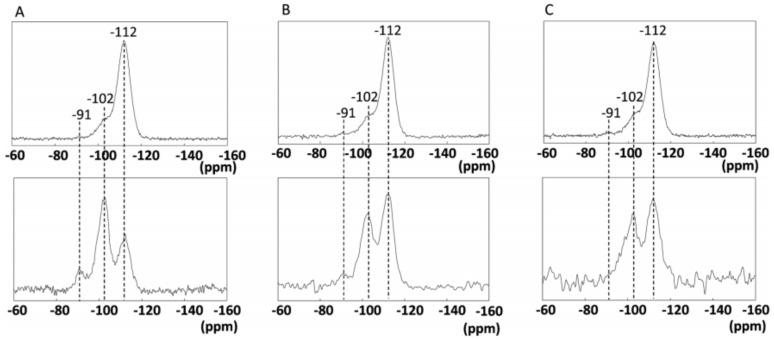
Single pulse (top) and H-CP ^29^Si MAS NMR spectra (bottom) of (**A**) acid-only-treated RUB-36, (**B**) Fe-IEZ-CDO, and (**C**) IEZ-Al-CDO. Reprinted with permission from ref. [65]. Copyright 2014, The Royal Society of Chemistry.

The use of a metal salt instead of a silylating agent to drive an interlayer expansion of a lamellar precursor causes the same shift in the first reflection. As shown in Figure 3D and Figure 11, acid treatment separates adjacent layers, allowing soluble silicon species from zeolite crystals to construct the interlayer-expanded structure. To confirm the coordination state of the incorporated iron species, single pulse (Figure 11, top) and H-CP ^29^Si MAS NMR techniques (Figure 11, bottom) are used to analyze (A) acid-only-treated RUB-36, (B) Fe-IEZ-CDO, and (C) IEZ-Al-CDO (see Figure 11A–C) [65,83,84]. For H-CP ^29^Si MAS, in particular, the peaks with the chemical shift around −112 ppm are attributed to the Q^3^ species [Si(OSi)_3_Fe or Si(OSi)_3_OH]. In the series formed by acid-only-treated RUB-36, IEZ-Al-CDO, and Fe-IEZ-CDO, the iron species content increases, whereas the signal with the chemical shift at −91 ppm [Q^2^, Si(OSi)_3_(OH)_2_] decreases. This result suggests that, in the presence of iron species, the linking sites are occupied by -Fe(OH)_2_- instead of by Si(OH)_2_ groups alone.

### 3.5. Investigation of Interlayer Expansion with UV-vis and X-ray Photoelectron Spectroscopy

**Figure 12:**
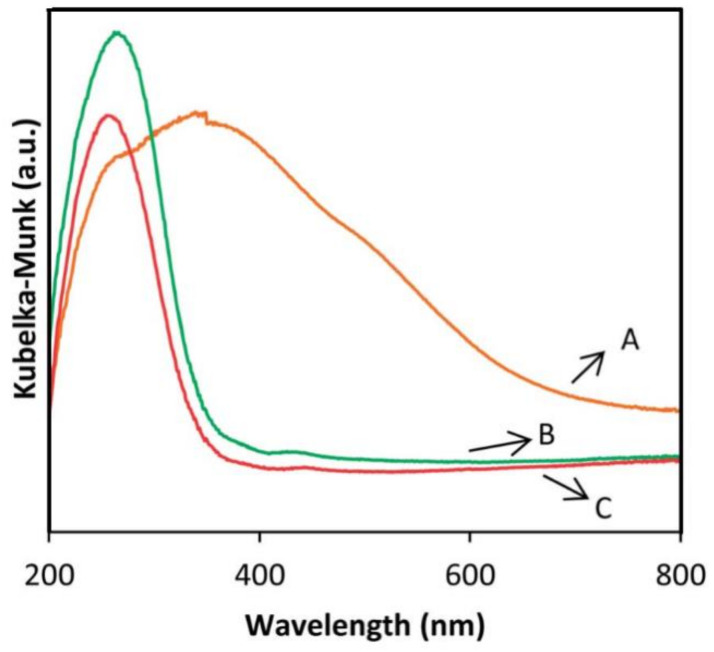
UV-vis spectra of (**A**) Fe_2_O_3_-ZSM-5, (**B**) Fe-IEZ-CDO, and (**C**) Fe-IEZ-Al-CDO. Reprinted with permission from ref. [65]. Copyright 2014, The Royal Society of Chemistry.

UV-vis absorption spectroscopy has been used to confirm that the metal atom is indeed incorporated at isolated sites. The original precursors produce no clear peaks because no metal is present [49]. However, once metal species are incorporated, the UV-vis spectra reflect the environment of these metal species [49,65]. Figure 12 compares the UV-vis spectra of Fe-IEZ-CDO, Fe-IEZ-Al-CDO, and normal Fe_2_O_3_-ZSM-5. Both Fe-IEZ-CDO and Fe-IEZ-Al-CDO contain one major absorption peak, representing the isomorphous substitution of Fe species in the framework [85,86]. On the contrary, normal Fe_2_O_3_-ZSM-5 produces a broad adsorption spectrum around 400 nm [87,88,89], indicating that Fe species in Fe-IEZ-CDO and Fe-IEZ-Al-CDO should be isolated.

**Figure 13:**
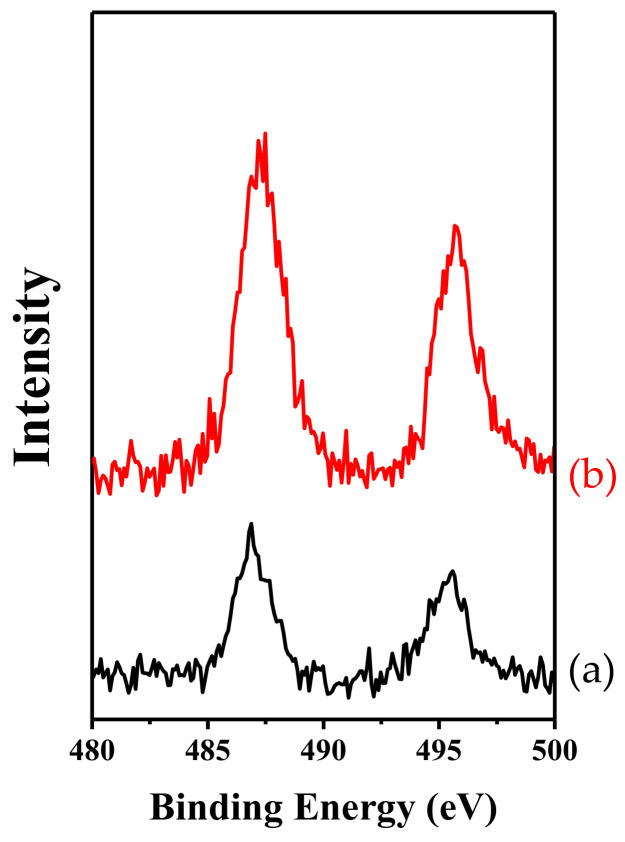
Sn 3*d*_5/2_ and 3*d*_3/2_ spectra of (**a**) Sn-IEZ-COK-5 and (**b**) Sn-IEZ-MFS. Reprinted with permission from ref. [55]. Copyright 2015, Elsevier.

X-ray photoelectron spectroscopy (XPS) is an important surface analysis technique. It not only provides information on molecular structure and the atomic valence state for chemical research, but also provides information on elemental composition, chemical state, and molecular structure. Figure 13 shows Sn 3*d*_5/2_ and 3*d*_3/2_ spectra for Sn-IEZ-COK-5 and Sn-IEZ-MFS, giving binding energies of 487.3 and 495.6 eV, respectively, which exceeds that of SnO_2_ crystals (485.8 and 494.4 eV, respectively). This phenomenon can be monitored by the isolated tin species in the zeolite framework [90,91,92,93].

## 4. Possible Mechanism

**Figure 14:**
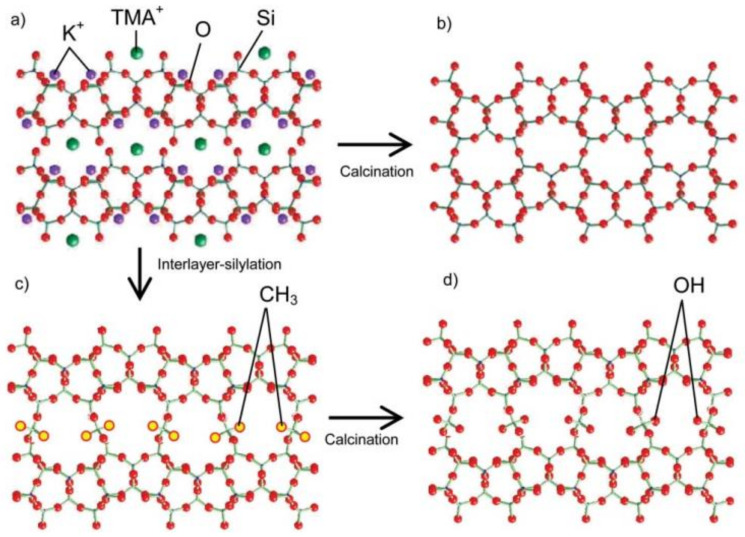
Possible schemes for the formation of the interlayer-expanded structure by the intercalation of DCDMS molecules: (**a**) PLS-1, (**b**) CDS-1, (**c**) IEZ-PLS-1, and (**d**) IEZ-CDS-1. Reprinted with permission from ref. [47]. Copyright 2007, Royal Society of Chemistry.

The above results allow us to propose a mechanism for constructing new zeolite structures by interlayer expansion of a layered precursor. A typical example is the formation of IEZ-PLS-1 and IEZ-CDS-1 from PLS-1, as shown in Figure 14 [47], The layered silicate PLS-1 has the chemical formula K_1.3_[(CH_3_)_4_NOH]_1.7_Si_18_O_40_(OH)_4_] Generally, 3D CDS-1 zeolites can be obtained by calcinating the PLS-1 lamellar precursor while condensing hydroxyl groups between adjacent layers. CDS-1 (Si_36_O_72_, CDO) consists of two 8-MR along the (001) and (010) directions. After silylation with DCDMS, 3D crystals of IEZ-PLS-1 are obtained with Si(CH_3_)_2_ groups incorporated. After calcination at 500 °C in air, the methyl groups attach to the interlayer silicon atoms and turn into hydroxyl groups. The sample obtained is denoted as IEZ-CDS-1. The interlayer silylation of CDS-1 increases from 8 to 10 MR along (020) direction.

**Figure 15:**
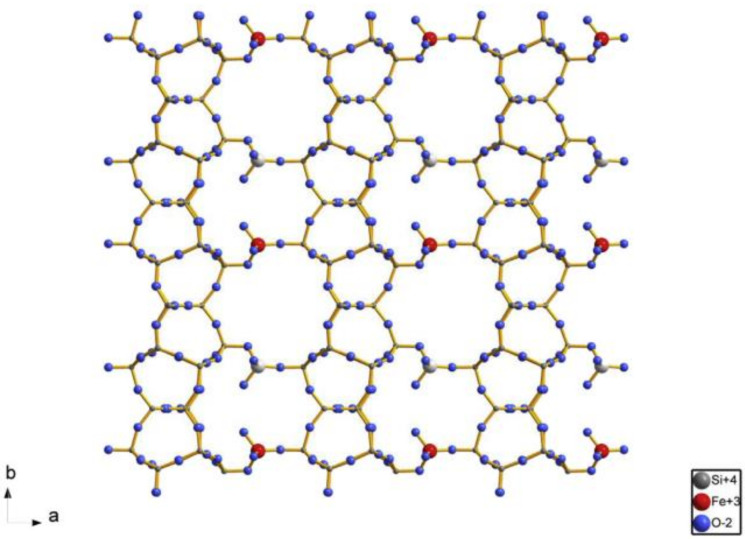
Projection along [001] of the framework of Fe-IEZ-CDO showing schematically the metal centers at every linker site. Reprinted with permission from ref. [66]. Copyright 2016, Elsevier.

The introduction of metal atoms at the linker sites with metal salts is reminiscent of similar formation mechanisms as the silylation progresses [65,94]. Taking Fe-IEZ-CDO as an example, the FeCl_3_ agents insert irons species connecting the silicon species of adjacent layers to form Fe-IEZ-RUB-36. After calcination, the irons species convert into -O-Fe(OH)_2_-O- groups and the samples convert into Fe-IEZ-CDO. Figure 15 shows clearly the detail in Fe-IEZ-CDO samples, with the link across the interlayer space clearly shown [51]. As opposed to the CDO structure, the linker -O-Fe(OH)_2_-O- groups attach to the interlay silicon atoms. The interlayer expansion thus increases of the layer distance, which increases the microporous volume and allows extra-atoms to insert themselves at the linker sites.

## 5. Catalytic Properties

### 5.1. Acidity Characterization

**Figure 16:**
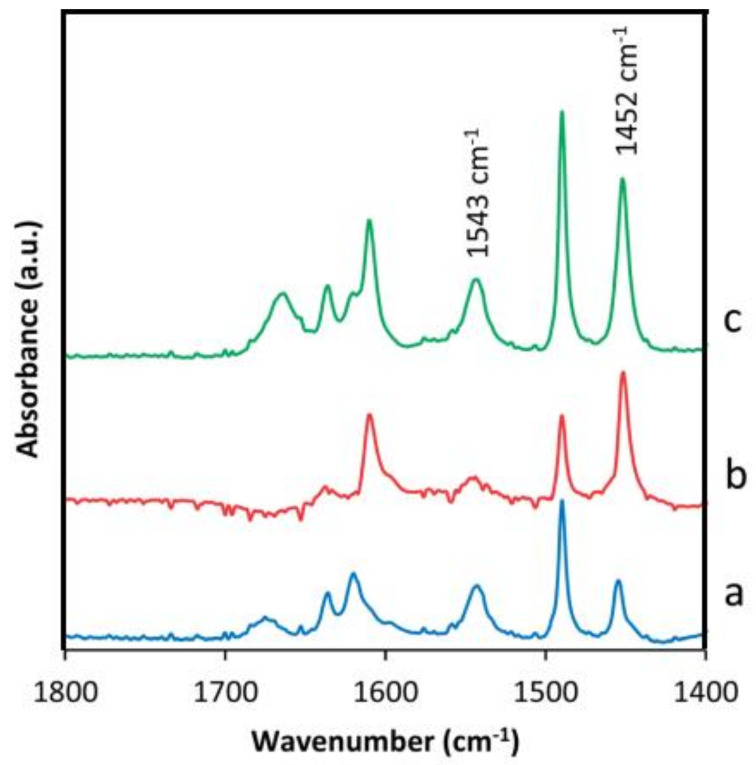
Different Py-FTIR spectra of adsorbed pyridine on (**a**) Al-CDO, (**b**) Fe-IEZ-CDO, (**c**) Fe-IEZ-Al-CDO. Reprinted with permission from ref. [65]. Copyright 2014, The Royal Society of Chemistry.

The acidity of the materials changes significantly after inserting metal atoms. The Lewis-Bronsted acidity is determined by Fourier-transform infrared spectroscopy of pyridine (Py-FTIR). All catalysts are detected in 10 mg sample tablets with a special shape. Figure 16 shows the difference spectra after adsorption of pyridine. The adsorption bands at 1455–1450, 1480–1490 and 1550–1545 cm^−1^ correspond to pyridine adsorbed at Lewis-Bronsted acid sites [76,95,96]. Compared with the original 3D crystals, the intensity associated with Lewis-acid sites increases for IEZ samples, which contributes to the incorporation of metal species. Compared with Al-CDO, Fe-IEZ-CDO, and Fe-IEZ-Al-CDO in Figure 16, the absorbance increases significantly due to the contribution of Fe and Al species incorporated into the precursor layer.

### 5.2. Catalytic Measurements

The IEZ material has larger pores than the 3D structure obtained by direct calcination and is expected to offer better catalytic activity [97,98,99,100,101]. Table 6 exhibits catalytic activity and selectivity in oxidation of cyclohexene over titanosilicates. IEZ-Ti-MWW has been widely accepted as an efficient catalyst in cyclohexene oxidation with H_2_O_2_ [102,103,104,105]. It is difficult for cyclohexene with a large dynamic diameter to penetrate the 10-MR window in 3D MWW. Expanding the interlayer space is the most effective way to make the Ti sites in the supercage serve as active sites for oxidizing large-volume substrate molecules. The phenomenon also occurs in Ti-CDO and IEZ-Ti-CDO materials [50]. For oxidizing cyclohexene, Ti-CDO samples have low conversion because of the inaccessibility for cyclohexene. Conversely, IEZ-Ti-CDO exhibits excellent activity.

**Figure 17:**
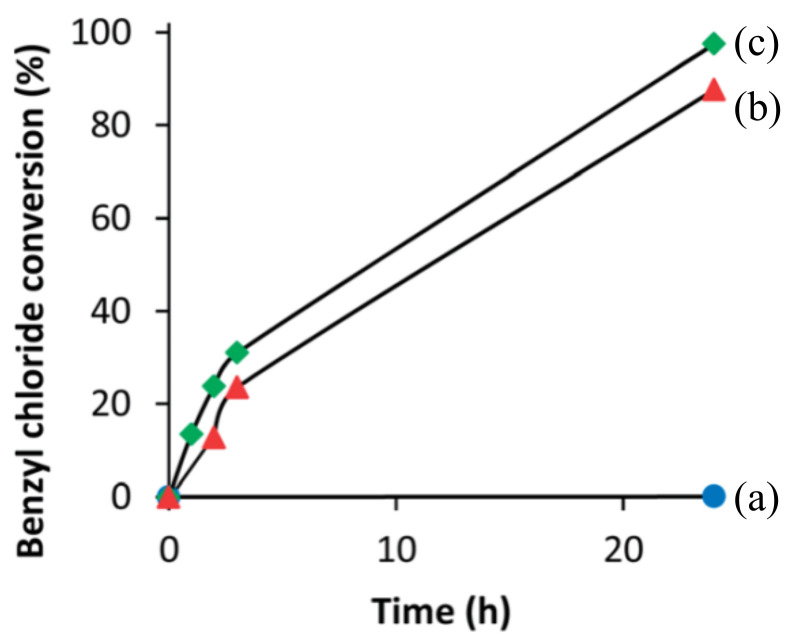
Benzyl chloride conversion in benzylation of toluene as a function of time at 403 K for (**a**) IEZ-Al-CDO, (**b**) Fe-IEZ-CDO, and (**c**) Fe-IEZ-Al-CDO. Reprinted with permission from ref. [65]. Copyright 2014, Royal Society of Chemistry.

In addition, the incorporation of metal species strongly increases the catalytic activity because the metal atoms provide active centers. The catalytic activity of expanded CDO materials is investigated in alkylation of toluene with benzyl chloride [65]. As shown in Figure 17, IEZ-CDO is not active for the alkylation reaction. Notably, Fe-IEZ-CDO and Fe-IEZ-Al-CDO offer significant benzyl chloride conversion. This result confirms that the incorporated Fe atoms play an important role in catalyzing this reaction.

### 5.3. Adsorption of Metal Cations

The IEZ materials have also been discussed for increased adsorption of metal cations (such as Ce) and enhanced activity. Roth and co-workers studied the MWW zeolite [106] and found the expansion of the interlayer distance increases would be accessible to these surface cavities and thus allow diffusion of ions. The IEZ-MWW samples form adsorbed twice as many cerium ions compared with the same Al content as MCM-22 (Table 7). The abundant adsorption of cerium species of Ce/IEZ-MWW samples enables higher CO oxidation. These results confirm the IEZ materials provide great potential opportunities in the catalytic applications.

## 6. The ADOR Synthesis of New Zeolites

**Figure 18:**
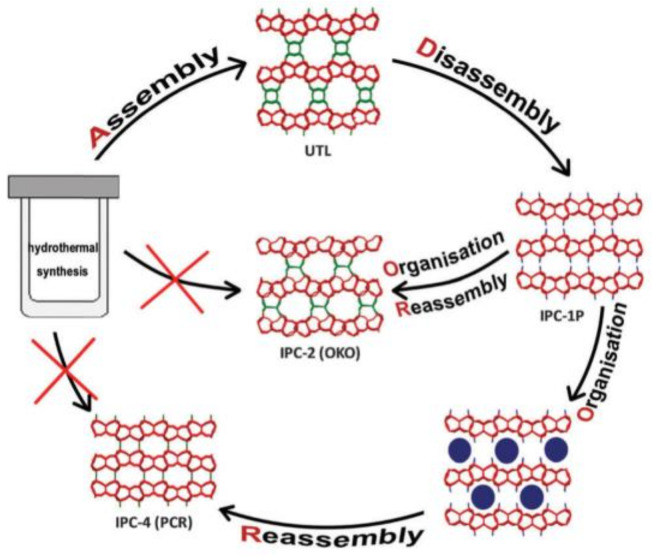
The ADOR method in a cycle scheme demonstrating the mechanism for the synthesis of two novel zeolites, IPC-2 (OKO) and IPC-4 (PCR), in which of them have not been sofa prepared by direct hydrothermal synthesis. The interlay bonds are highlighted in green, and the terminal silanol groups in blue. Reprinted with permission from ref. [8]. Copyright 2015, Royal Society of Chemistry.

Besides the direct synthesis of layered zeolites, a special top and down methodology named ADOR is designed (assembly-disassembly-organisation-reassembly) [107,108,109]. Typically, the new structures PCR and OKO have been synthesized by this method (Figure 18). In order to obtain the lamellar precursors, IPC-1P, original zeolite UTL samples are treated in a Walker-type multi-anvil apparatus at 1GPa with heating 200 °C for 2 min. Through controlled organisation of IPC-1P, one can prepare a series of materials (IPC-2, IPC-4, IPC-6, IPC-7) [109,110,111,112,113]. When the organization agents refer to the silane type (diethoxydimethylsilane, DMDOES), the silanol groups connect to the surface of IPC-1P and interlayer expand the distance. After intercalation of organizing agent, the obtained OKO framework, IPC-2, possess 12 and 10-ring channels. This method has been successfully applied for other zeolite SAZ [114].

## 7. Conclusions

This review briefly summarizes the novel zeolite structures constructed from the lamellar precursor zeolites MWW, RUB-36, PREFER, Nu-6(1), COK-5, and PLS-1 via interlayer expansion, with silicon species or metal species inserted and bonded to Si atoms in the lamellar layer. A variety of measurements could confirm that functional linker sites connected to Si atoms in the lamellar layer. The resulting materials with ordered crystalline structure have larger pore windows and tunable wettability, which is beneficial for varying the catalytic function. The incorporation of metal atoms provides an extra source of catalysts for large molecules. The use of interlayer-expansion agents to connect layered silicates proves to be a general route for these novel zeolite structures. This method and the stable materials obtained could have important industrial applications in the near future.

## Figures and Tables

**Table 1:** The published zeolite synthesis via topotactic condensation of layered silicates. Reprinted with permission from ref. [22]. Copyright 2012, Schweizerbart Science Publishers.

Layered Silicate	Zeolite	Zeolite Framework Type, Degree of Order	Reference
EU-19	EU-20	CAS, disordered	[23]
MCM-65 (as made)	MCM-65 (calcined)	CDO, fair ordered	[24]
PLS-1	CDS-1		[25]
UZM-13, UZM-17, UZM-19	UZM-25	CDO, well ordered	[26]
RUB-36, RUB-38, RUB-48	RUB-37		[27]
PLS-4	/		[28]
PREFER	Ferrierite	FER, well ordered	[29]
PLS-3	CDS-3		[28]
MCM-22 precursor	MCM-22	MWW, well ordered	[30]
ITQ-1	ITQ-1 (calcined)		[31]
EMM-10-p	EMM-10	MWW, disordered	[32]
NU-6(1)	Nu-6(2)	NSI, well ordered	[15]
RUB-39	RUB-41	RRO, well ordered	[17,18,19]
RUB-18	RUB-24	RWR, fair ordered	[7,20]
RUB-15	Silica-Sodalite	SOD, fairly ordered	[33]
IPC-1P	IPC-4	PCR, well ordered	[34]

**Table 2:** Published synthesis routes involving IEZs of layered silicates.

Layered Silicate	Zeolite Framework ^1^	IEZ	Insertion Agent	Name in Refs.	Reference
RUB-36	CDO	IEZ-CDO	DEDMS, DCDMS	COE-4	[52]
		M-IEZ-CDO ^2^	FeCl_3_, Metal-acetylacetone	M-COE-4 M-JHP-2	[49,51]
Ti-RUB-36		IEZ-Ti-CDO	DHDMS	Ti-COE-4	[50]
Al-RUB-36		IEZ-Al-CDO	DEDMS, DCDMS	Al-COE-4	[53]
		Fe- IEZ-Al-CDO	FeCl_3_	Al-COE-4/Fe	[51]
PLS-1		IEZ-CDS-1	DCDMS	IEZ-2	[47]
Nu-6(1)	NSI	IEZ-NSI	DEDMS	IEZ-Nu-6	[54]
COK-5	MFS	IEZ-MFS	DCDMS	COE-6	[40]
		M-IEZ-MFS	Metal-acetylacetone ^2^	M-COE-6 ^2^,	[55]
Me-MWW precursor ^3^	MWW	IEZ-Me-MWW	DEDMS	IEZ-MWW	[38]
Ti-MWW precursor		IEZ-Ti-MWW	DEDMS	Ti-YNU-1	[48]
PREFER	FER	IEZ-FER	DEDMS	IEZ-FER	[56]
PLS-3		Ti-IEZ-FER	TiCl_4_	Ti-ECNU-8	[57]

^1^ The framework refers to the framework of calcined layered zeolites. ^2^ M = Fe, Sn, Zn, etc. ^3^ Me = Al, Ga, Fe, etc.

**Table 3:** Textural parameters of RUB-36 and the related zeolites.

Name	Chemical Composition	Surface Area (m^2^/g)	Micro Volume (cm^3^/g)	2*θ* degree ^1^	*d* Spacing ^2^ (Å^2^)	Reference
RUB-36	(C_6_H_16_N)_4_(H_4_Si_36_O_76_)	40	<0.01	8.14	11.2	[52]
CDO	Si_36_O_72_	288	0.12	9.76	9.2	[52]
IEZ-RUB-36	Si_20_O_38_(CH_3_) _4_	238	0.063	7.53	-	[52]
IEZ-CDO	Si_20_O_38_(OH) _4_	350	0.131	7.92	11. 74	[52]
Fe-IEZ-CDO	Si_19.14_Fe_0.86_O_38_(OH)_4_	423	0.156	7.90	11.7	[51]
Sn-IEZ-CDO	Si_38.6_Sn_1.4_O_76_(OH)_8_	362	0.17	7.90	11.7	[51]
Al-CDO	-	231	0.09	8.14	9.2	[65]
IEZ-Al-CDO	-	364	0.135	7.90	11.74	[65]
Fe-IEZ-Al-CDO	-	389	0.136	7.89	11.7	[65]
Ti-CDO	-	189	0.09	8.13	-	[50]
IEZ-Ti-CDO	-	294	0.13	7.90	-	[50]

^1^ The 2*θ* degree refers to the layer-related diffraction, i.e., the 2*θ* degree of the first peak.^2^ The *d*-spacing data are derived from the first reflection in the powder XRD patterns.

**Table 4:** *d* spacing determined by HRTEM for 3D zeolites after calcination and IEZ samples. Reprinted with permission from ref. [36]. Copyright 2008, American Chemical Society.

Structure Type	*d* Spacing (Å)		
	*hkl*	3D Zeolites	IEZ Samples
MWW	100	12.3	12.7
	001	25.3	28.2
FER	200	9.8	12.1
	020	7.3	7.2

**Table 5:** Physicochemical properties of MFS and IEZ-MFS under different conditions. Reprinted with permission from ref. [40]. Copyright 2015, Elsevier.

Zeolite	DCMDS ^1^ (g/g of COK-5)	Si/Al Ratio ^2^	Contact Angle ^3^	Contact Angle before Calcination^4^
MFS	--	24	27°	28°
IEZ-MFS-0	0	74	27°	56°
IEZ-MFS-0.185	0.185	40	13°	109°
IEZ-MFS-0.375	0.375	36	41°	120°
IEZ-MFS-0.830	0.830	40	31°	69°

^1^ Samples were treated with DCDMS in 1M HCl at 180 °C for 24 h. ^2^ Si/Al ratios were measured by ICP technique. ^3^ The contact angles were measured for water on the surface. ^4^ The contact angles before calcination refers to the contact angles of COK-5, IEZ-COK-5-0, IEZ-COK-5-0.185, IEZ-COK-5-0.375, IEZ-COK-5-0.830.

**Table 6:** Catalytic activity and selectivity in oxidation of cyclohexene over titanosilicates.

Catalyst	Conversion	TOF (h^−1^)	Selectivity		Reference
			Epoxide	Others	
Ti-MWW ^1^	8.1	/	35.0	65.0	[26]
IEZ-Ti-MWW ^1^	21.2	/	90.8	9.2	
Ti-CDO ^2^	4.2	19.1	/	/	[40]
IEZ-Ti-CDO ^2^	14	63.9	/	/	

^1^ Reaction conditions: 60 °C, 2 h, 50 mg catalyst, 10 mL acetonitrile as solvent, 10 mmol cyclohexene, 10 mmol of H_2_O_2_. ^2^ Reaction conditions: 60 °C, 4 h, 50 mg catalyst, 10 mL methanol as solvent, 10 mmol cyclohexene, 10 mmol of H_2_O_2_.

**Table 7:** Basic properties of the studied materials. Reprinted with permission from ref. [108]. Copyright 2014, Royal Society of Chemistry.

Zeolite	Si/Al, XRF	BET, m^2^g^−1^	Ce(La), wt%	BAS ^1^ μmol g^−1^	LAS ^2^ μmol g^−1^
MCM-22	13	436	-	664	46
IEZ-MWW	13	531	-	621	112
Ce/MCM-22 ^3^	13	-	0.40	714	95
Ce/IEZ-MWW ^3^	13	-	0.88	357	70

**^1^** BAS refers to Bronsted acid site concentrations. ^2^ LAS refers to Lewis acid site concentrations. ^3^ The cerium here is adsorption by ion-exchange.

## Data Availability

Data available in a publicly accessible repository.

## Sample Availability

Samples of the compounds are not available from the authors.
